# Mixed Cations Enabled Combined Bulk and Interfacial Passivation for Efficient and Stable Perovskite Solar Cells

**DOI:** 10.1007/s40820-023-01085-7

**Published:** 2023-04-30

**Authors:** Pengfei Wu, Shirong Wang, Jin Hyuck Heo, Hongli Liu, Xihan Chen, Xianggao Li, Fei Zhang

**Affiliations:** 1 School of Chemical Engineering and Technology, Tianjin University, Tianjin, 300072 People’s Republic of China; 2 Collaborative Innovation Center of Chemical Science and Engineering (Tianjin), Tianjin, 300072 People’s Republic of China; 3 Department of Chemical and Biological Engineering, Korea University, 145 Anam-Ro, Seongbuk-Gu, Seoul, 17104 Republic of Korea; 4 SUSTech Energy Institute for Carbon Neutrality, Department of Mechanical and Energy Engineering, Southern University of Science and Technology, Shenzhen, Guangdong 518055 People’s Republic of China

**Keywords:** Alternating-cation-interlayer, Bulk defects, Interfacial passivation, Perovskite solar cells

## Abstract

Here, we report a mixed GAI and MAI (MGM) treatment method by forming a 2D alternating-cation-interlayer (ACI) phase (n = 2) perovskite layer on the 3D perovskite, modulating the bulk and interfacial defects in the perovskite films simultaneously, leading to the suppressed nonradiative recombination, longer lifetime, higher mobility, and reduced trap density. Consequently, the devices’ performance is enhanced to 24.5% and 18.7% for 0.12 and 64 cm^2^, respectively. In addition, the MGM treatment can be applied to a wide range of perovskite compositions, including MA-, FA-, MAFA-, and CsFAMA-based lead halide perovskites, making it a general method for preparing efficient perovskite solar cells. Without encapsulation, the treated devices show improved stabilities.

**Supplementary Information:**

The online version contains supplementary material available at 10.1007/s40820-023-01085-7.

## Highlights

- A mixed GAI and MAI (MGM) treatment modulating the bulk and interfacial defects simultaneously is reported.
- The devices’ performance is enhanced to ~24.5% in 0.12 cm^2^ and ~18.7% in 64 cm^2^ with improved stability.
- The MGM treatment can be applied to a wide range of perovskite compositions.

**Supplementary Information:**

The online version contains supplementary material available at 10.1007/s40820-023-01085-7.

## Graphical abstract

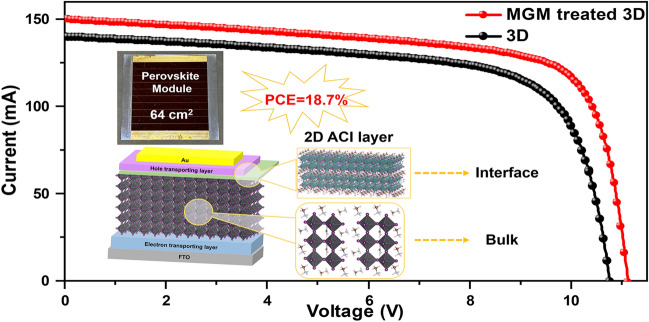

## Introduction

Due to their outstanding optoelectronic features, low cost, and easy solution process, hybrid halide perovskites are the most potential next-generation semiconductor material for solar cells [1–3]. Mainly, perovskite solar cells (PSCs) have attained power conversion efficiencies (PCEs) of over 25% in single-junction devices and over 30% in tandem devices, compared with commercial silicon solar cells [4]. Nevertheless, it should be noted that the stability of PSCs is still inferior to commercial solar cells. This results from the high density of defects at grain boundaries and interfaces, leading to the severe degradation and short lifetime of PSCs [5, 6].

To surmount these deficiencies, several strategies have been reported [7–13]. Specifically, depositing 2D perovskite layers on the top surface of 3D perovskites is one of the efficient solutions for forming a 2D/3D perovskite heterojunction, which can effectively passivate interfacial defects and restrain ion migration [14–16]. The 2D/3D perovskite heterojunction has been successfully integrated into regular n-i-p structured PSCs. The pure 2D perovskite (n = 1) layers were inserted between the 3D perovskite films and hole transporting layers (HTLs) to boost device efficiency and stability simultaneously [17, 18]. However, the pure 2D perovskite (n = 1) layers often represent low out-of-plane charge transport, act as a charge-extraction barrier, and inhibit device operation [19–21].

The high-n phase 2D-perovskite interfacial passivation layer (n ≥ 2) with higher out-of-plane charge transport (including hole and electron) is a more promising alternative to creating 2D/3D heterojunction, showing much-improved stability and higher PCE [8, 22–24]. However, 2D/3D heterojunctions with high-n phase 2D-perovskite (n ≥ 2) are rarely reported [8, 22–25]. In addition, high-n phase 2D-perovskite (n ≥ 2) passivation layers only suppress the defects in the interface and do not modulate charge defects in the bulk. Moreover, lattice mismatching between the amino group from bulky cations of the 2D passivation layers and 3D perovskite lattices is one of the key factors determining the device performance [26].So it is urgent to develop some new passivation layers modulating the interface and bulk defects simultaneously with lattice matching to boost performance and stability further.

Here, we developed 2D alternating-cation-interlayer (ACI) phase (n = 2) perovskite layer on the 3D perovskite (Fig. S1) by the Mixed guanidinium iodide(GAI) and methylammonium iodide(MAI) (MGM) treatment method. For the suitable ionic radius and plentiful ammonium in molecular structure, guanidinium cation(GA^+^) could provide more hydrogen bonds to coordinate with the surrounding halides and stabilize the lattice [27]. ACI (n = 2) is one type of 2D perovskite with the chemical formula of (GA)(MA)_2_Pb_2_I_7_ (n = 2), which is stabilized by the ordering of GA^+^ and methylammonium cation(MA^+^) alternatively in the interlayer space [28]. This 2D perovskite has a better out-plane charge transfer property (Fig. S2) than that of the customarily used RP-phase PEA_2_PbI_4_ (PEA:2-phenylethylammonium) and DJ-phase BDAPbI_4_ (BDA: 1,4-butanediammonium), due to its narrower optical bandgap (1.99 eV) and reduced van der Waals gaps [27, 28]. Besides the 2D ACI layer formation at the top surface, the GA^+^ and MA^+^ can penetrate bulk perovskite through grain boundaries due to the suitable ionic radius (Table S1) [29, 30], passivating both the bulk and interfacial defects. On the other hand, the 2D ACI /3D heterostructure facilitates energy level alignment to improve the charge transfer. Consequently, the device based on MGM treatment showed a PCE of ~ 24.5% in 0.12 cm^2^ and ~ 18.7% in 64 cm^2^. The optimized PSCs using the MGM treatment also featured increased PCEs based on an extensive range of perovskite compositions. The MGM treatment-based PSCs showed improved stability compared to 3D perovskite devices.

## Experimental Section

### Materials

All chemical reagents were used as received without any purification. guanidinium iodide (GAI, 99.5%), poly(3,4-ethylenedioxythiophene)-poly(styrenesulfonate (PEDOT:PSS), 4-tert-butyl pyridine (tBP, > 96%) and lithium salt-bis(trifluoromethane)sulfonimide imide (LiTFSI), 2,2′,7,7′-tetrakis-(N,Ndi-p-methoxyphenylamine)-9,9′-spirobifluorene (spiro-OMeTAD, 99.5%) were pursued from Xi’an Polymer Light Technology, China. SnO_2_ colloidal solution (15 wt% in H_2_O) was purchased from Alfa Aesar. Acetonitrile (ACN, 99.8%), N,N-dimethylformamide (DMF, 99.8%), dimethyl sulfoxide (DMSO, 99.8%), isopropanol (IPA) and chlorobenzene (CB, 99.9%) were purchased from Sigma-Aldrich. PbI_2_ (99.99%), formamidinium iodide (FAI, 99.5%), methylammonium iodide (MAI, 99.5%), methylammonium chloride (MACl, 99.5%) and florine doped tin oxide (FTO) were purchased from Yingkou Advanced Election Technology Co., Ltd.

### Device Fabrication

#### Standard Devices

*FAPbI*_*3*_*-based device fabrication*: FTO was sequentially cleaned with deionized water, IPA, and ethanol for 15 min, respectively. Before use, the FTO/glass substrates were treated with ultraviolet ozone for 10 min. Then, the FTO/glass substrates were spin-coated with a thin layer of SnO_2_, diluted in water at a 1:5 ratio, at 3,000 rpm for 30 s, followed by annealing at 150 °C for 30 min. The 3D FAPbI_3_ film fabrication was deposited according to the previous report [31]. Then, a certain concentration of GAI and MAI was dissolved in IPA and spin-coated on the surface of the perovskite at 5,000 r.p.m for 30 s, followed by annealing for 5 min at 100 °C. Subsequently, The spiro-OMeTAD solutions were prepared by dissolving 72.3 mg of spiro-OMeTAD in 1 mL CB, with the addition of 28.8 µL of tBP and 17.5 µL of LiTFSI (520 mg dissolved in 1 mL of ACN). The solution was spun at 4,000 rpm for the 20 s. For the top electrode, 80 nm gold was thermally deposited at an evaporation rate of 0.2 ~ 0.8 A s^−1^.

*MAPbI*_*3*_*-based,* FA_0.85_MA_0.15_PbI_2.55_Br_0.45_*-based and FA*_*0.85*_*MA*_*0.1*_*Cs*_*0.05*_*PbI*_*2.9*_*Br*_*0.1*_*-based device fabrication*: Devices were fabricated on conductive FTO-coated glass substrates. FTO/glass was sequentially cleaned with deionized water, IPA, and ethanol for 15 min Before use, the FTO/glass substrates were treated with ultraviolet ozone for 10 min. a compact TiO_2_ blocking layer was deposited by spray pyrolysis of 10-mL IPA solution containing 0.86 mL titanium diisopropoxide bis(acetylacetonate) solution (75% in 2-propanol, Sigma-Aldrich) and 0.57 mL acetylacetone at 450 °C in air. On top of this layer, mesoporous titanium dioxide was formed by spin-coating 30-nm-sized nanoparticles (Dyesol 30NRD, Dyesol) diluted in ethanol (1:6 w/w) at 4,000 rpm for 20 s. The MAPbI_3_ precursor solution was prepared by mixing 1.55 M PbI_2_ and1.55 M MAI in DMF:DMSO = 4:1. The spin-coating procedure was performed at 2,000 rpm for 10 s followed with 6,000 rpm for 30 s. After entering the second step, 120 μL of CB was dropped on the spinning substrate in the last 15 s. Thereafter, the substrate was put onto a hotplate for 60 min at 100 °C. The FA_0.85_MA_0.1_Cs_0.05_PbI_2.9_Br_0.1_ precursor solution was prepared by mixing 1.30 M PbI_2_ with excess 5% PbI_2_, 0.14 M PbBr_2_, 1.2 M FAI, 0.16 M MABr, and 0.08 M CsI in DMF: DMSO = 4:1. The spin-coating procedure was performed at 2,000 rpm for 10 s followed by 6,000 rpm for 20 s. After entering the second step, 120 μL of CB was dropped on the spinning substrate in the last 15 s. Thereafter, the substrate was put onto a hotplate for 60 min at 100 °C. The FA_0.85_MA_0.15_PbI_2.55_Br_0.45_ precursor solution was prepared by mixing 1.35 M Pb^2+^ (PbI_2_ and PbBr_2_) in the mixed solvent of DMF and DMSO, the volume ratio of DMF/DMSO is 4:1. After entering the second step, 120 μL of CB was dropped on the spinning substrate in the last 15 s. Thereafter, the substrate was put onto a hotplate for 60 min at 100 °C. For MGM treatment, a certain concentration of GAI and MAI in IPA solution was spin-coated onto the perovskite film at 5,000 rpm for 30 s with subsequent annealing for 5 min at 100 °C. Subsequently, The spiro-OMeTAD solutions were prepared by dissolving 72.3 mg of spiro-OMeTAD in 1 mL CB, with the addition of 28.8 µL of tBP and 17.5 µL of LiTFSI (520 mg dissolved in 1 mL of ACN). The solution was spun at 4,000 rpm for 20 s. For the top electrode, 80 nm gold was thermally deposited at an evaporation rate of 0.2 ~ 0.8 A s^−1^.

#### Module Devices

The modules were fabricated following the same procedure as the process for the small area devices except for the P1, P2, and P3 scribing with a laser scriber (ND:YVO_4_, 355 nm, 6 W). The large area FTO substrates were first patterned with a P1 line to isolate the bottom electrodes and were washed in an ultrasonication bath. The SnO_2_, perovskite and spiro-OMeTAD were sequentially deposited following the coating procedures as described above. The prepared stacks were scribed with P2 lines to allow a series connection between the cells and deposited Au electrodes. Finally, P3 scribes were patterned on the evaporated films to isolate the top electrodes.

### Measurements and Characterizations

X-ray diffraction (XRD) patterns were collected by Mini Flex 600 with a Cu-Kα1 x-ray source. The fluorescence was excited by a laser at 465 nm, and a HORIBA Scientific FlouroMax-4 recorded the steady-state photoluminescence (PL) spectra with an excitation wavelength of 500 nm. Surface and cross-sectional scanning electron microscopy (SEM) images were conducted by field emission scanning electron microscope (S-4800, Hitachi). The out-plane mobilities were tested by the similar reported SCLC method [20]. A Thermo Fisher ESCALAB-250Xi carried out X-ray photoelectron spectroscopy (XPS) with an Al-Kα X-ray source gun type. The ultraviolet photoemission spectroscopy (UPS) was performed on a photoelectron spectrometer Kratos Analytical, ESCALAB-250Xi, with the He(I) excitation at 21.22 eV. The time-resolved PL spectra (TRPL) were recorded by a HORIBA Fluorolog-3 spectrometer equipped with a 450 W Edinburgh Xe900 Xenon lamp as the exciting light source; Bruker Dimension Icon AFM probed atomic force microscopy (AFM) and the Kelvin probe force microscopy. The incident light-to-electron conversion efficiency (IPCE) was tested using the E0201a IPCE testing system (Chinese Academy of Science) and calibrated by a silicon solar cell; PSCs were placed under the AM1.5 solar simulator (100 mW cm^−2^); *J-V* curves were tested and recorded by the Keithley 2400 source meter with the scanning speed of 100 mV s^−1^, and the effective area was 0.12 cm^2^. HI = (PCE_Reverse_-PCE_Forward_)/PCE_Reverse_, where PCE_Reverse_ and PCE_Forward_ represent the PCE obtained from reverse and forward scans, respectively. All the measurements were performed in air at room temperature with < 40% humidity. The contact angles were tested using a JC 2000D contact angle instrument.

*TOF–SIMS test*: The ToF–SIMS analysis was conducted using a TOF.SIMS 5–100 instrument (ION-TOF GmbH, Müenster, Germany). During the data acquisition, a pulsed 30 kV Bi_3_^+^ primary ion beam with a pulse length of 13.5 ns was utilized to generate the secondary ions. The beam current of the primary ion beam was 30 nA-DC. The ToF–SIMS results were obtained from a 100 μm × 100 μm area on the sample surface. A sample bias of 2 kV accelerated the ejected secondary ions, so the secondary ions could gain enough kinetic energy to reach the SIMS detector. All the tracked secondary ions were positively charged monovalence fragments. A pulsed 20 V electron flood gun was applied during the data acquisition to compensate for the surface charge. A 500 V O_2_^+^ sputter ion beam was applied during the sputtering to remove the surface material. The beam current of the O_2_^+^ sputter beam was 80 nA-pulse. The O_2_^+^ sputter beam was rastered over a large 300 μm × 300 μm area to avoid the crater-edge effect that might interfere with the observations.

*TR test*: A pump-probe spectrometer (TA-100, Time-tech spectra) performed the TR test. A Ti: Sapphire amplifier (Astrella, Coherent) generates 800 nm light at a 1 kHz repetition rate. The fundamental pulse is split into two parts. One part is sent to an optical parametric amplifier (TOPAS, Lightconversion) for the pump wavelength generation at 2.58 eV. The pump is chopped at a frequency of 500 Hz and attenuated by neutral-density filter wheels. The other part of the fundamental pulse focuses on a sapphire crystal to generate a visible continuum (450–810 nm) used as the probe. The probe pulses are delayed in time concerning the pump pulses using a motorized translation stage mounted with a retro-reflecting mirror. The pump and probe are spatially overlapped on the surface of the sample. For TR measurement, the incident angle for the pump is around 0°, and the probe is around 45°.

## Results and Discussion

### Properties of Control and MGM-Treated Perovskite Thin Films

**Fig. 1:**
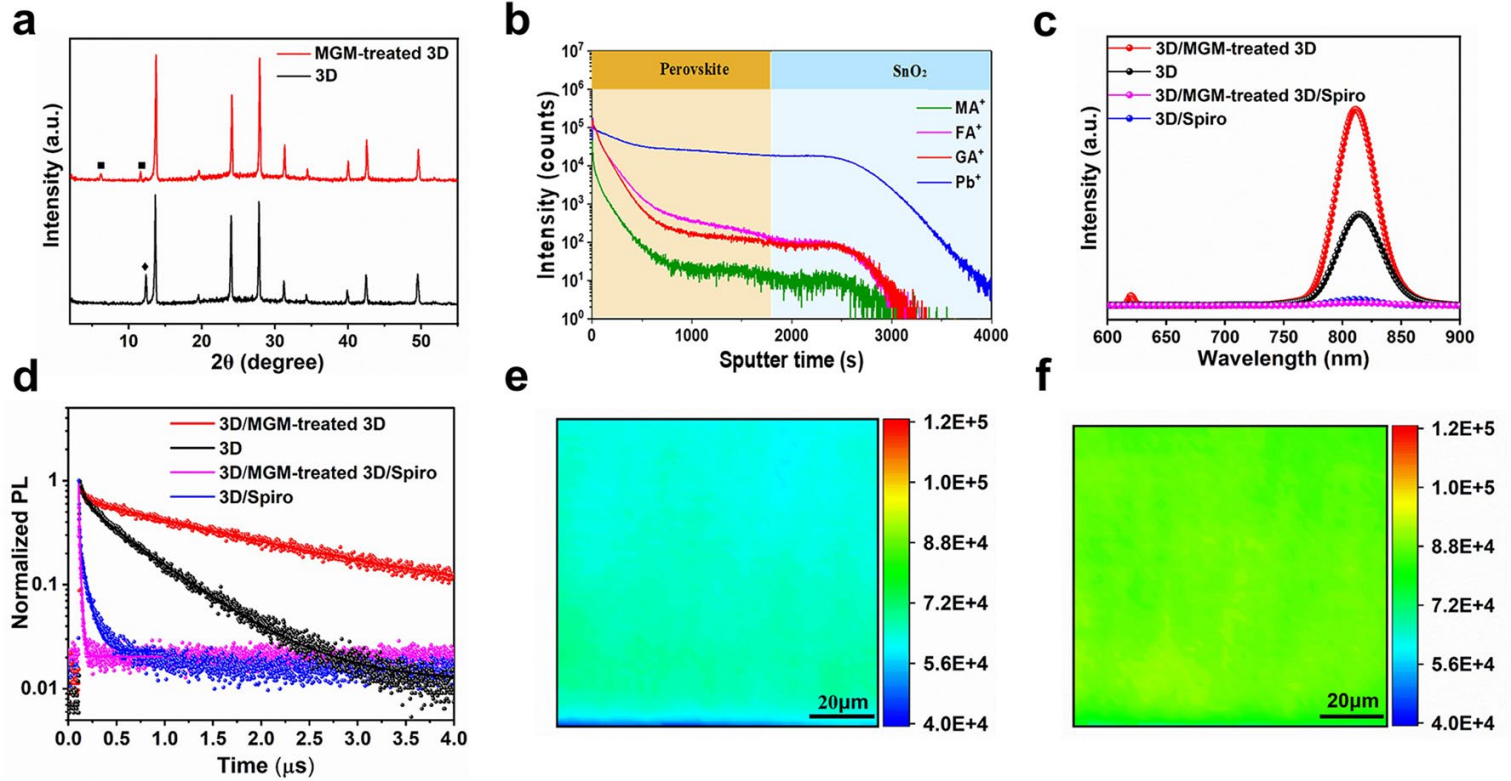
The properties of perovskite films. **a** XRD patterns of 3D and MGM-treated 3D film. **b** ToF–SIMS depth profiles of the MGM-treated 3D perovskite film deposited on the FTO/SnO_2_ substrate. **c** Steady-state PL spectra and** d** TRPL spectra of 3D film, MGM-treated 3D film, 3D/Spiro, MGM-treated 3D film/Spiro. PL mappings of **e** 3D film and **f** MGM-treated 3D film, respectively (scale bar: 20 μm)

Here, we did the mixed cations passivation treatment by spin-coating a GAI and MAI/IPA solution on top of the 3D FAPbI_3_ perovskite, followed by further annealing at 100 ℃ for 5 min. We first utilized thin-film X-ray diffraction (XRD) measurements of the corresponding perovskite films (Fig. 1a). Both films displayed intense peaks at ≈14° and ≈28°, corresponding to the (001) and (002) planes, respectively [32]. After MGM treatment was employed, the diffraction of the PbI_2_ phase was reduced. In contrast, two conspicuous peaks at≈6° and 11.6° (noted as square) emerged, corresponding to the formation of the ACI (n = 2) 2D perovskite structure (Fig. S3).

To confirm the distribution of the 2D ACI layer on the 3D perovskite film, the time-of-flight secondary ion mass spectrometry (ToF–SIMS) was performed. ToF–SIMS depth profiles and the corresponding 3D images of corresponding films are illustrated in Figs. 1b and S4a. The distribution of GA^+^ and MA^+^ were detected throughout the entire perovskite bulk and had particular penetration characteristics, as verified by the ToF–SIMS depth profiles shown in Fig. S4b–. The 2D ACI surface layer could suppress the interfacial defects. Moreover, the diffused GA^+^ and MA^+^ in 3D perovskite could passivate the bulk defects, including ionic vacancies and uncoordinated Pb and/or Pb clusters, and stabilize the perovskite structure resulting in enhanced charge transport and reduced ion migration [33, 34].

To better understand the effect of MGM treatment on carrier dynamics, steady-state photoluminescence (PL) and time-resolved photoluminescence (TRPL) decay measurements were conducted. Compared with 3D perovskite, the PL intensity of the MGM-treated perovskite film became stronger (Fig. 1c). In addition, there was a peak at 620 nm, referred to as 2D ACI (n = 2), indicating the formation of a 2D ACI interfacial passivation layer. The MGM-treated perovskite film had a longer decay time (τ_avg_) than the 3D film (Fig. 1d and Table S2). The increased PL intensity and the longer τ_avg_ exhibited reduced nonradiative recombination [35, 36]. Conversely, after spin-coating a Spiro-OMeTAD HTL on the perovskite film, the PL intensity of the MGM-treated 3D perovskite film had a lower fluorescence intensity and shorter lifetime than 3D film, indicating improved hole transport due to the suppression of the bulk and interfacial defects [37]. Further, the PL mapping (Fig. 1e-f) showed that the MGM-treated 3D perovskite film presented a more uniform and stronger PL emission than the control film at a large scale, consistent with the result of PL spectra. The ultrafast transient reflection (TR) spectroscopy (Fig. S5) also showed the existence of the 2D perovskite layer and the efficient exciton transfer from the 2D layer to the 3D perovskite layer.

To evaluate the effect of MGM treatment on the surface morphology of perovskite film, scanning electron microscopy (SEM) was performed, and the results are displayed in Fig. S6a, d. After the MGM treatment, the apparent grain size increased, and the grain boundaries reduced. The cross-sectional SEM images of the devices based on 3D film and MGM-treated 3D film are shown in Fig. S6b, e. Compared with 3D film, crystal grains of MGM treated 3D film across the complete 3D perovskite film were closely packed, which could increase the carrier transport and mitigate iodide ion diffusion at grain boundaries and improve the stability of PSCs [38]. The atomic force microscopy (AFM) was also used to investigate the surface morphologies of perovskite film with and without MGM treatment. As displayed in Fig. S6c, f, the root-mean-square roughness of the MGM-treated 3D film (26.3 nm)was lower than that of the 3D film (39.7 nm), delivering a smooth appearance after MGM treatment.

**Fig. 2:**
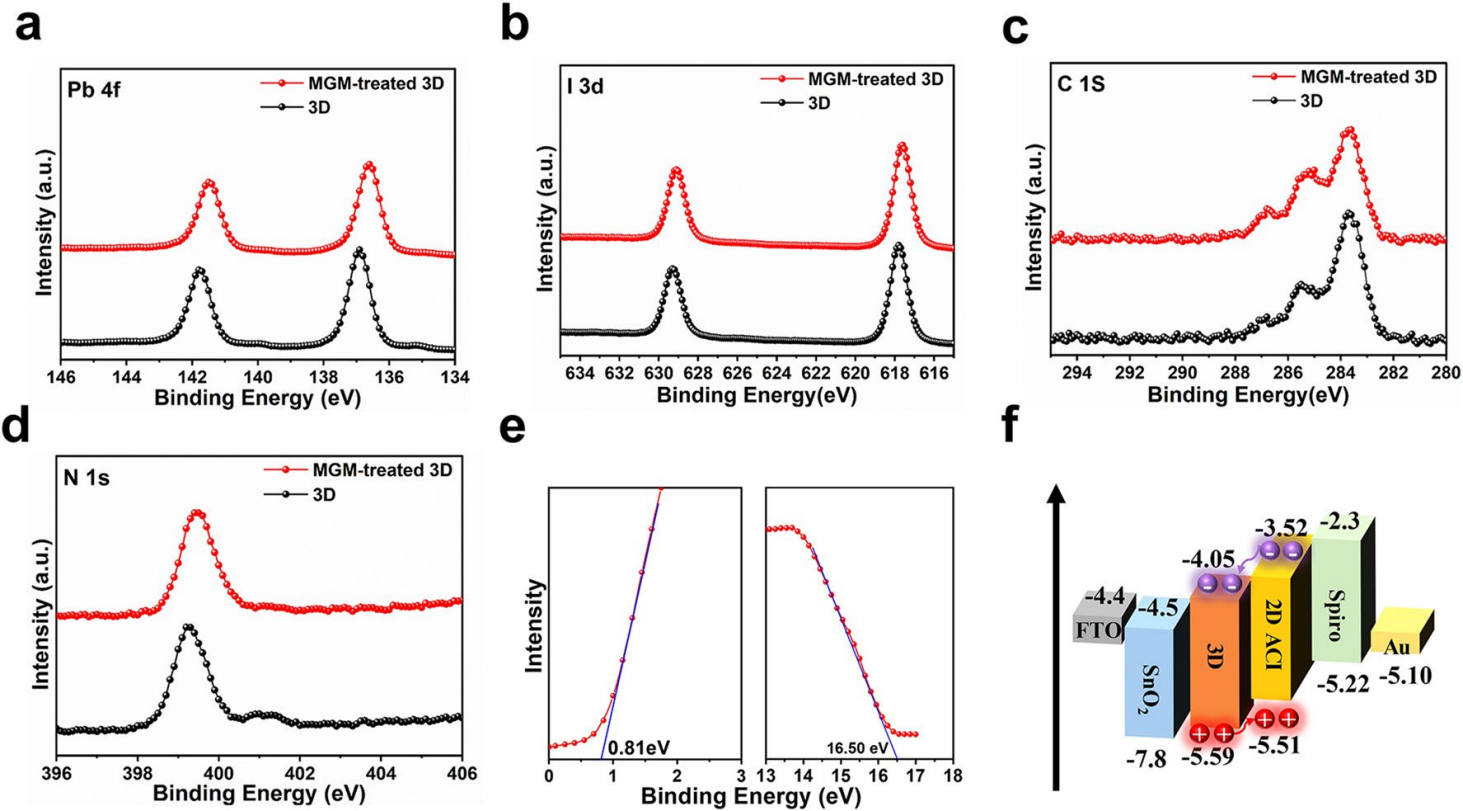
Interaction characterization and bandgap property. XPS spectra of **a** Pb 4*f*; **b** I 3*d*; **c** C 1*s*, and **d** N 1*s* of the 3D film and MGM-treated 3D film. **e** UPS energy spectrum of the MGM-treated 3D film in the cutoff region (right) and onset region (left). **f** Schematic energy-level diagram of the device

To further verify the formation of the 2D ACI perovskite layer, the XPS measurements were performed on 3D film and MGM-treated 3D film. As shown in the Pb 4*f* and I 3*d* spectra **(**Fig. 2a-b**)**, the position of the peak suggested apparent shifts to the lower binding energy after MGM treatment, as Pb 4*f* shifted from 136.8 and 141.7 eV to 136.5 and 141.4 eV, and I 3*d* shifted from 617.8 and 629.2 eV to 617.4 and 628.8 eV respectively, confirming the strong interactions between cations (GA^+^ and MA^+^) in ACI and [PbI_6_]^4−^ octahedra framework [39, 40]. In the C 1*s* spectra, the stronger peak at 286.7 eV was evidence of C = NH_2_^+^ of the GA^+^. **(**Fig. 2c**)**. The XPS of N 1*s* also exhibited a strong chemical interaction attributed to the GA group **(**Fig. 2d**)** [41, 42].

Ultraviolet photoelectron spectroscopy (UPS) measurement was performed to examine the perovskites' energy level. From the calibrated UPS spectra (Figs. 2e and S7) and the energy level diagrams **(**Fig. 2f**)**, the valence band maximum (VBM) of the MGM-treated 3D perovskite film had a better energy-lever alignment with other functional layers compared with 3D film. Thus, introducing a 2D ACI perovskite layer can elevate hole transfer from perovskite to Spiro-OMeTAD, reducing interface charge recombination and achieving a higher *V*_*OC*_ [14, 25].

**Fig. 3:**
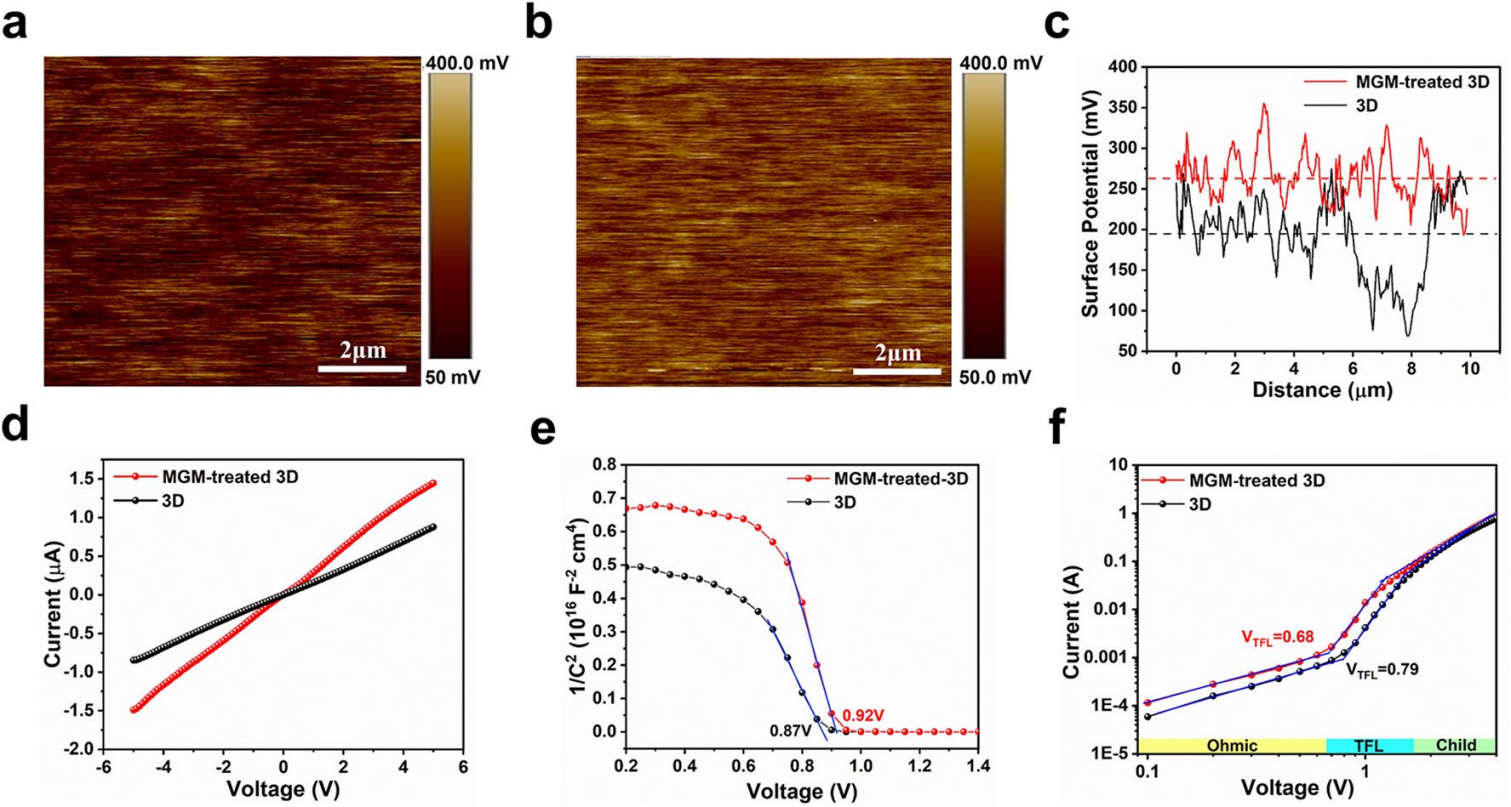
The film surface property.** a** KPFM images of 3D film. **b** KPFM images of MGM-treated 3D film. **c** The line profile of surface potential of MGM-treated 3D and 3D film. **d** *J–V* characteristics of MGM-treated 3D and 3D film under dark. **e** MS curves of the MGM-treated 3D and 3D film-based devices; the slope was obtained by fitting the linear part. **f** The dark *J–V* characteristics of hole-only devices with MGM-treated 3D and 3D film

Kelvin probe force microscopy (KPFM) was introduced to probe the effect of MGM treatment on the surface potential. The surface of the MGM-treated 3D perovskite film displayed a higher electronic chemical potential than that of 3D film, demonstrating a better hole extraction **(**Fig. 3a–c**).** The reason most likely arises from minor interfacial recombination defects and the perovskite film's slightly shallow work function (WF) with a 2D ACI layer [43, 44]. Additionally, the influence of MGM treatment on the electrical conductivity was investigated, as shown in Fig. 3d. Higher electrical conductivity was observed in MGM-treated 3D film, which originated from reduced surface defects and bulk defects of the 3D films [45]. Furthermore, the Mott-Schottky test **(**Fig. 3e**)** was also performed to study the effect of MGM treatment on the devices’ built-in potential (*V*_*bi*_). The *V*_*bi*_ was obtained according to the following Eq. 1 [46]:

1 $$\frac{1}{{C}^{2}}=\frac{2}{{A}^{2}{N}_{q}{\varepsilon }_{0}\varepsilon }\left({V}_{bi}-\mathrm{V}-\frac{2{k}_{B}T}{q}\right)$$

where *A* is the active device area, *N*_*q*_ is the carrier concentration, *V*_*bi*_ is the built-in potential, *V* is the applied bias, and *C* is the capacitance. The *V*_*bi*_ value was enhanced from 0.87 V for the 3D film-based device to 0.92 V for the MGM-treated 3D film-based device. The higher *V*_*bi*_ was ascribed to the better energy level alignment brought about by 2D ACI passivation, which was an internal driving force to separate the photo-generated carriers for the improved *V*_*OC*_ of the device [47].

To further quantify the trap density in the perovskite films, we utilized the space-charge-limited-current (SCLC) technique. The corresponding *J-V* curves for hole-only devices are shown in Fig. 3f. The hole-only device is with the structure of ITO/PEDOT: PSS/perovskite/MoO_x_/Al. The hole trap density (*N*_*t*_) can be given from Eq. 2 [48]:

2 $${V}_{\mathrm{TFL}}=\frac{e{N}_{t}{L}^{2}}{{2\varepsilon }_{0}\varepsilon }$$

where *e* is the elementary charge, *L* is the perovskite film thickness, *ε* is the relative dielectric constant of perovskite, *ε*_*0*_ is the vacuum permittivity, and *V*_*TFL*_ is the trap-filling voltage. The *V*_*TFL*_ of pristine perovskite film and optimal perovskite film with MGM treatment was 0.79 V and 0.68 V, corresponding to the *N*_*t*_ of 1.08 × 10^16^ and 9.16 × 10^15^ cm^−3^, respectively. This result demonstrated a reduced trap density with MGM passivation.[49]. In addition, the hole mobility of the polycrystalline perovskite film was calculated via Mott-Gurney Eq. 3 [50]:

3 $${J}_{D}=\frac{9{\varepsilon }_{0}\varepsilon \mu {V}_{b}}{8{L}^{3}}$$

where *J*_*D*_, *ε*, *ε*_*0*_, *µ*, *V*_*b*_, and *L* are the current density, the relative dielectric constant, the vacuum permittivity, the mobility of perovskite film, applied voltage, and the thickness of the perovskite film, respectively. Consequently, the hole mobility of perovskite film with MGM treatment was 0.263 cm^2^ V^−1^ s^−1^, which was higher than that of 3D perovskite film (0.196 cm^2^ V^−1^ s^−1^) [20, 51]. We also checked the electron trap density and electron mobility using the SCLC method based on "electron-only" geometry (ITO/SnO_2_/perovskite/PCBM/Au, Fig. S8). The electron trap density of perovskite film with MGM treatment (~ 3.1 × 10^16^ cm^−3^) was lower than the pristine perovskite film (~ 6.5 × 10^16^ cm^−3^). Whereas, the electron mobility of perovskite film with MGM treatment (~ 1.89 cm^2^ V^−1^ s^−1^) was higher than the pristine perovskite film (~ 1.10 cm^2^ V^−1^ s^−1^), which is consistent with the TRPL and KPFM results as well as the higher *Voc* and *FF* in solar cells.

### Photovoltaic Performance of Solar Cells

**Fig. 4:**
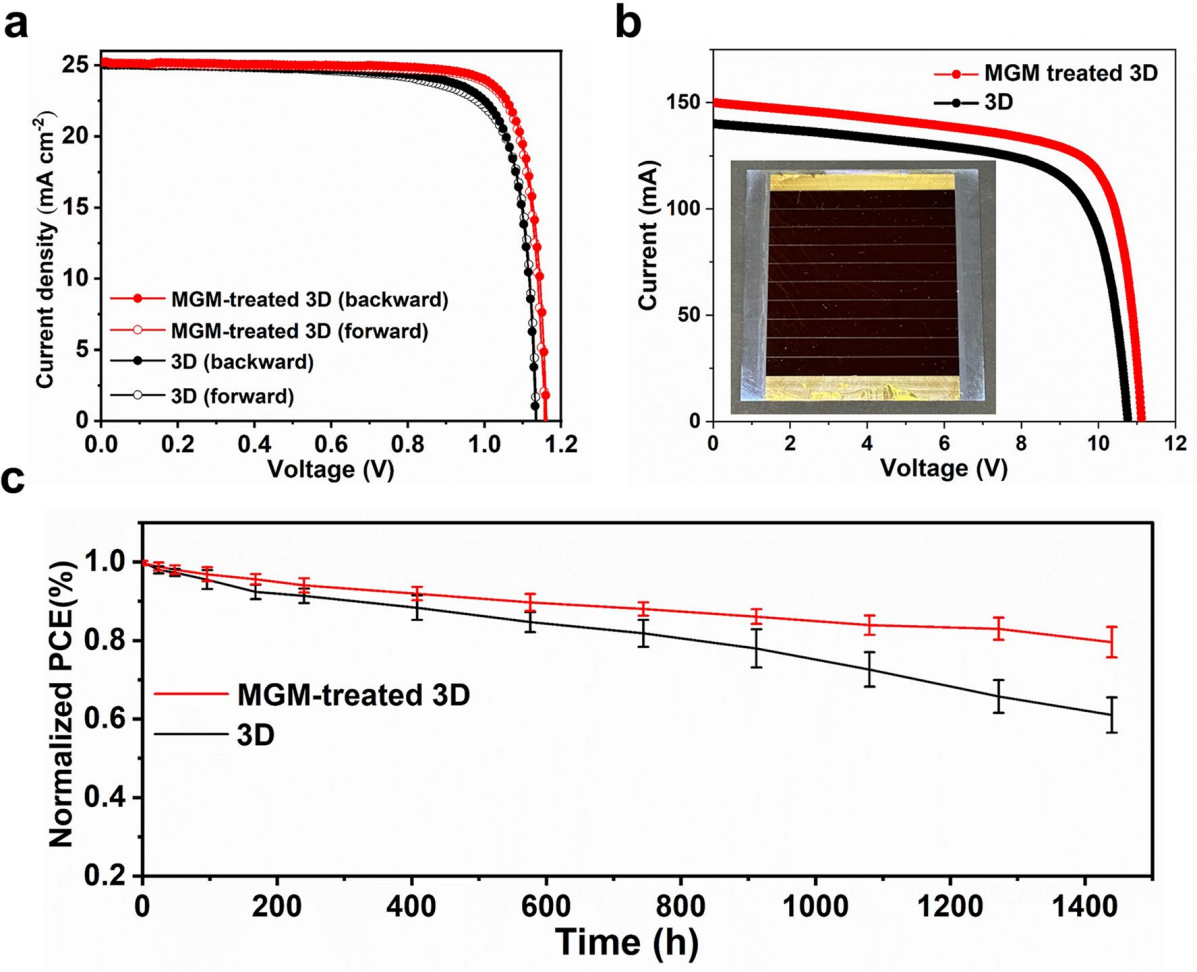
The device performance. **a** *J–V* curves of the champion small area devices **b** *J–V* curves of champion mini-module devices in standard simulated 100 mW cm^−2^ (AM 1.5G) illumination.** c** Long-term stability of 3D film and MGM-treated 3D film-based devices (6 devices in each condition) storage in air (RH = 30%-50%)

To examine the influence of MGM treatment on the photovoltaic properties, we fabricated perovskite devices with different mixed cations concentrations based on the n–i–p structure of FTO/SnO_2_/perovskite/spiro-OMeTAD/Au (Fig. S9). After optimization, the density–voltage (*J–V*) curves and corresponding photovoltaic parameters of champion devices are shown in Fig. 4a and listed in Table S3. The champion PCE of the devices was significantly enhanced from 22.6% for the 3D film-based device to 24.5% with the MGM treatment, mainly arising from the *V*_*OC*_ increase (from 1.13 to 1.16 V) along with an improved *FF* (from 80 to 84%). The PCE improvement was also consistent with defect passivation and efficient charge transport. Additionally, MGM-treated 3D film-based device exhibited a lower hysteresis index (HI) than the pristine device, which was probably due to the passivation of bulk and interface simultaneously and efficiently preventing the ion diffusion [52, 53]. Moreover, the statistical distribution of the PCE of PSCs suggested the superior reproducibility of devices with MGM-treated 3D film (Fig. S9). The integrated current densities estimated from the incident photon to electron conversion efficiency (IPCE) spectra (Fig. S10) and stabilized power output (SPO) (Fig. S11) of the corresponding PSCs were all in good agreement with *J*_*sc*_ and the PCE obtained from the *J–V* curves, respectively. Compared with commonly used ammonium salts treatment including phenethylammonium iodide (PEAI) and butylammonium iodide (BAI) and, devices with MGM treatment showed a higher PCE due to the simultaneous bulk and interfacial passivation and enhanced out-plane charge transfer (Fig. S12). In addition to the single FA-based perovskite composition, we also found that the PCEs of MAPbI_3_-, FA_0.85_MA_0.15_PbI_2.55_Br_0.45_-, and FA_0.85_MA_0.1_Cs_0.05_PbI_2.9_Br_0.1_-based PSCs with MGM treatment were enhanced compared to corresponding control PSCs (Fig. S13). Moreover, we also fabricated PSC mini-modules (64 cm^2^ aperture area, 60.4 cm^2^ active area) by the same treatment, and an increased 18.7% efficiency with good reproducibility (Fig. S14) from 16.3% was achieved (Fig. 4b). The enhanced *V*_*OC*_ and *FF* was due to the improved electron mobility and reduced defect density, making it a general method for preparing efficient PSCs with different areas. Dark *J–V* measurements were carried out to study the leakage current (*J*_*0*_) caused by the recombination of carriers at the interface. As shown in Fig. S15, the 3D film displayed a *J*_*0*_ value of 1.17 × 10^−6^ mA cm^−2^. In contrast, the *J*_*0*_ of PSCs with MGM treatment was reduced to 9.14 × 10^−8^ mA cm^−2^. The lower *J*_*0*_ generated less charge recombination along with larger *V*_*OC*_ in PSCs [46, 54].

We further checked the PSC stability using the protocols described in a recent consensus report [55]. Figure 4c shows the shelf stability (ISOS-D-1 stability) of unencapsulated PSCs under an ambient atmosphere with relative humidity (RH) of 30% − 50% at room temperature. The 3D film-based PSCs dropped to ~ 60% of their original PCE after 1440 h, while the MGM-treated 3D film-based PSCs still retained ∼80% of their initial PCE. The water contact angle measurement was carried out to verify the effect of 2D ACI on resistance to water (Fig. S16). The MGM-treated 3D perovskite film showed a larger contact angle than the 3D film, exhibiting a better hydrophobicity. To further verify the practical application of MGM-treated 3D film-based PSCs, all the devices were placed in the natural environment (Fig. S17a, ISOS-O-2 stability). The 3D film-based devices gradually go from black to pale yellow, demonstrating the severe degradation (Fig. S17b). On the contrary, the MGM-treated 3D film-based devices had few noticeable difference, revealing the excellent weather resistance due to the bulk and surface passivation. Lastly, we checked the operational stability of unencapsulated PSCs under one sun by maximum power point (MPP) tracking at ~ 30 °C in Ar (ISOS-L-1 stability). The MGM-treated 3D film-based devices retained ~ 97% of the initial PCE after > 100 h (Fig. S18), in contrast, the 3D film-based devices dropped to ~ 91% of their original PCE after 100 h.

## Conclusions

In summary, our study demonstrated a general mixed cations passivation method that simultaneously modulates the bulk and interfacial defects. Compared with pristine perovskite film, the formed 2D ACI (n = 2) interfacial layer had a better energy-level alignment with 3D perovskite film to maximize the charge transport. On the other hand, both the GAI and MAI could permeate across the surface into the bulk, contributing to the simultaneous passivation of the bulk and interfacial defects. As a result, the device based on MGM treatment showed a PCE of ~ 24.5% in 0.12 cm^2^ and ~ 18.7% in 64 cm^2^ with application in a wide range of perovskite compositions, including MA-, FA-, MAFA- and CsFAMA-based lead halide perovskites. Notably, the devices based on MGM treatment also showed enhanced stability to 3D film-based PSCs due to suppressed nonradiative recombination, longer lifetime, higher mobility, reduced trap density, and increased hydrophobicity. This work offers a feasible strategy to design efficient and stable perovskite solar cells by applying new high-n 2D (n ≥ 2) /3D heterojunctions along with bulk passivation.

### Supplementary Information

Below is the link to the electronic supplementary material.

Supplementary file1 (PDF 1173 kb)
