# Supplementary material for: Mixed Cations Enabled Combined Bulk and Interfacial Passivation for Efficient and Stable Perovskite Solar Cells
Source: Nanomicro Lett. 2023 Apr 30;15:114. doi: 10.1007/s40820-023-01085-7 (PMC10149427; doi:10.1007/s40820-023-01085-7)
Supplement: Supplementary file 1 — Supplementary file1 (PDF 1173 kb) [file 40820_2023_1085_MOESM1_ESM.pdf]

Supporting Information for

Mixed Cations Enabled Combined Bulk and Interfacial Passivation for Efficient and Stable Perovskite Solar Cells

Pengfei Wu[1, 2], Shirong Wang, [1, 2, *] Jin Hyuck Heo[3, *] ,Hongli Liu[1, 2], Xihan Chen[4], Xianggao Li[1, 2], Fei Zhang[1, 2, *]

[1] School of Chemical Engineering and Technology, Tianjin University, Tianjin 300072, P. R. China

[2] Collaborative Innovation Center of Chemical Science and Engineering (Tianjin), Tianjin 300072, P. R. China

[3] Department of Chemical and Biological Engineering, Korea University, 145 Anam-Ro, Seongbuk-Gu, Seoul 17104, Republic of Korea

[4] SUSTech Energy Institute for Carbon Neutrality, Department of Mechanical and Energy Engineering, Southern University of Science and Technology, Shenzhen, Guangdong, 518055 P. R. China

*Corresponding authors. E-mail:: wangshirong@tju.edu.cn (Shirong Wang); live2000jin@gmail.com (Jin Hyuck Heo); fei_zhang@tju.edu.cn (Fei Zhang)

Supplementary Figures and Tables

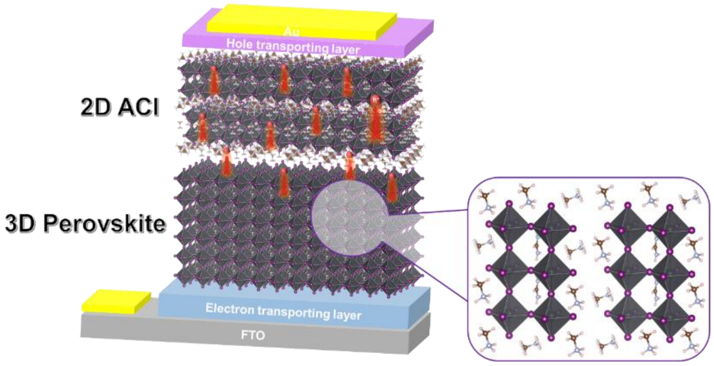

Fig. S1 Schematic depiction of the proposed 2D ACI/3D hybrid perovskite structure

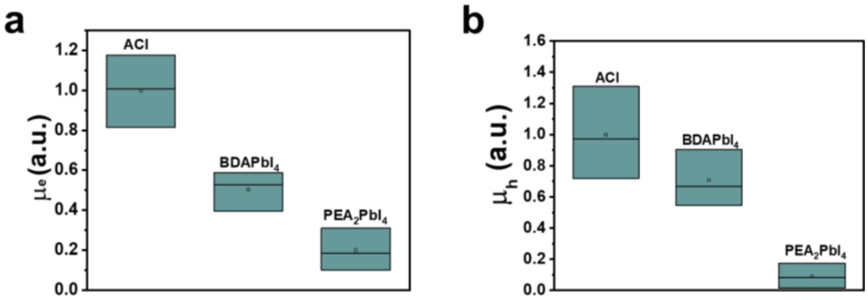

Fig. S2 Statistical comparison of the normalized out-of-plane hole **a** and electron **b** mobilities by SCLC (3 devices in each condition)

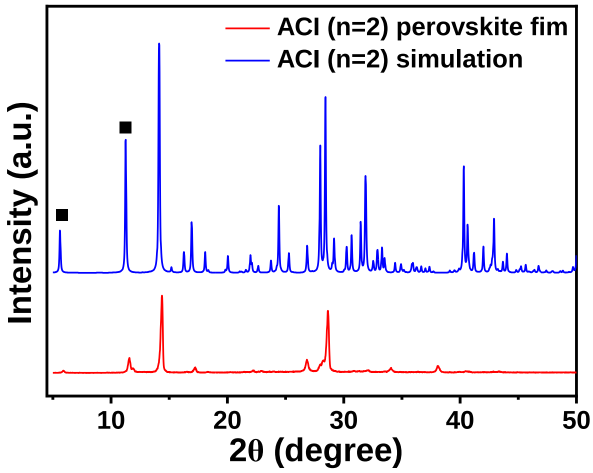

Fig. S3 XRD patterns of experimental and simulated 2D ACI (n=2)

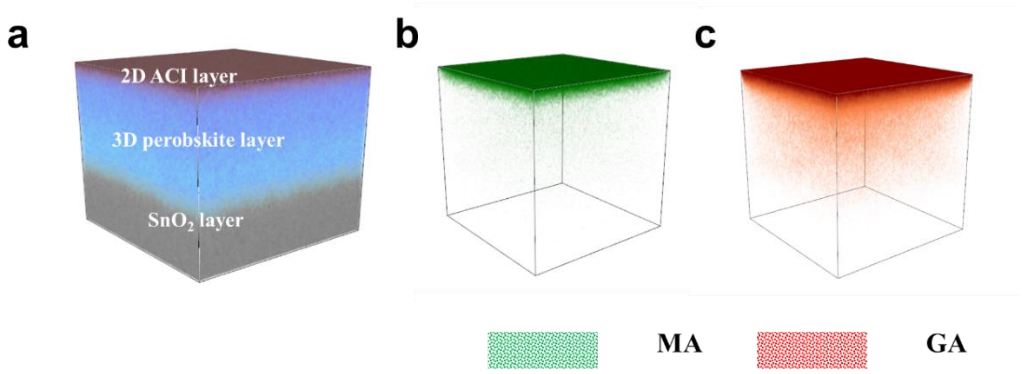

Fig. S4 a ToF-SIMS 3D images of the MGM-treated 3D film deposited on the FTO/SnO_2 substrate. **b** Side-view 3D images of MA distribution. **c** Side-view 3D images of GA distribution

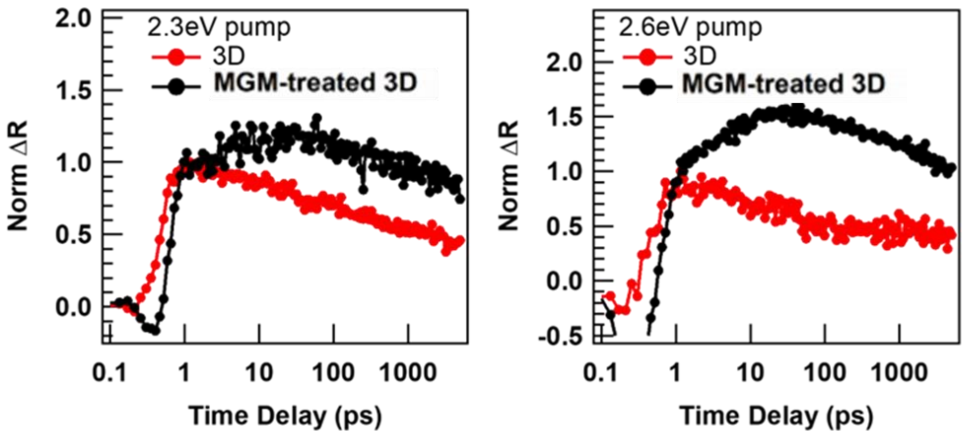

Fig. S5 Transient reflection (TR) comparison of the control and MCP-treated perovskite thin films. For control films, the TR kinetics rises within 1ps, and the decay starts, indicating a normal carrier diffusion/recombination process. For MGM-treated films, the TR kinetics exhibit additional rise kinetics from 1 to 30 ps, which is a characteristic of the charge transfer process

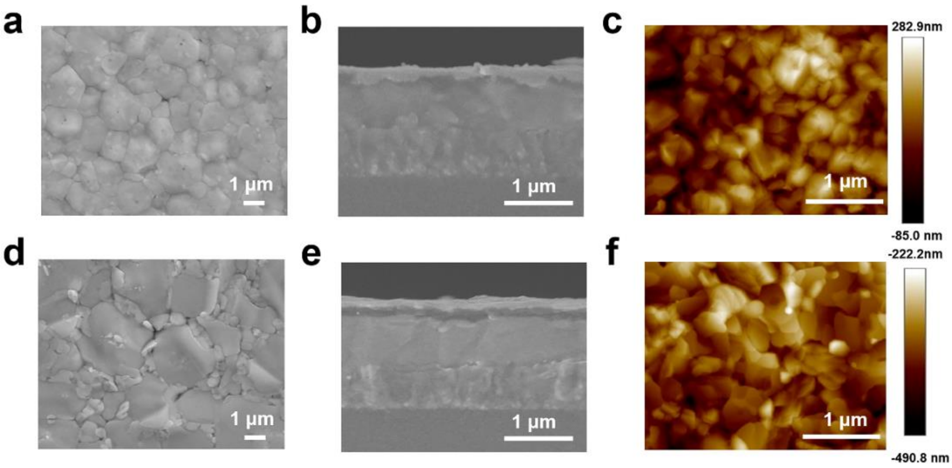

Fig. S6 a Top-view SEM images of 3D film. **d** Top-view SEM images of MGM-treated 3D film. The scale bar is 1μm. **b** Cross-sectional SEM image of 3D film. **e** Cross-sectional SEM image of MGM-treated 3D film. The scale bar is 1 μm. **c** Surface AFM images of 3D film. **f** Surface AFM images of MGM-treated 3D film. The scale bar is 1 μm

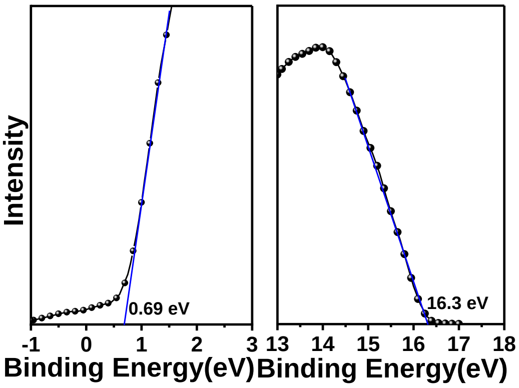

Fig. S7 UPS energy spectrum of the 3D film in cutoff region (right) and onset region (left)

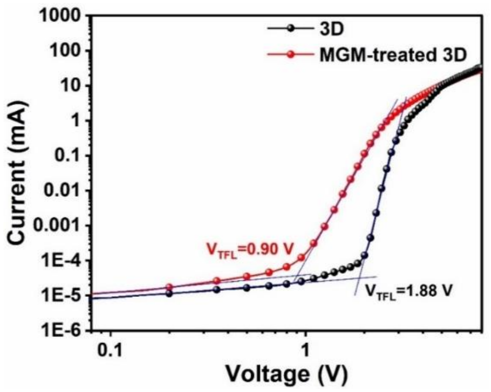

Fig. S8 Comparison of the control and MGM-treated perovskite thin films by space-charge-limited current measurement based on "electron-only" geometry (ITO/ SnO_2/perovskite/PCBM/Au)

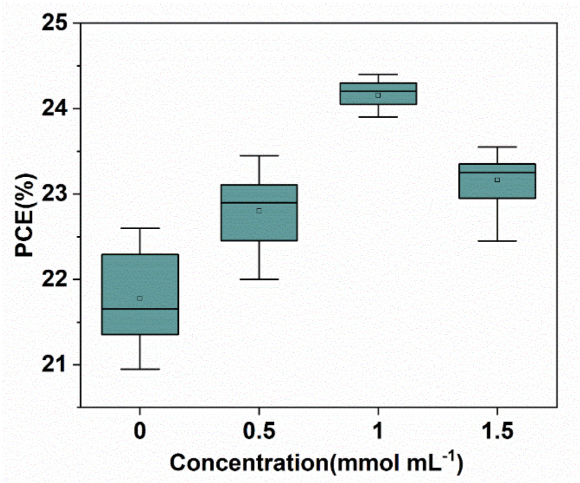

Fig. S9 The statistical distribution of the PCE of the devices with different MGM concentrations. Each condition has 20 devices

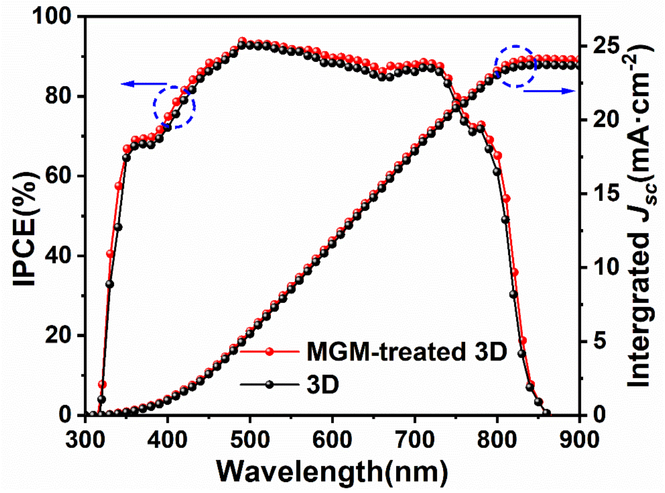

Fig. S10 IPCE of 3D film and MGM-treated 3D film-based devices

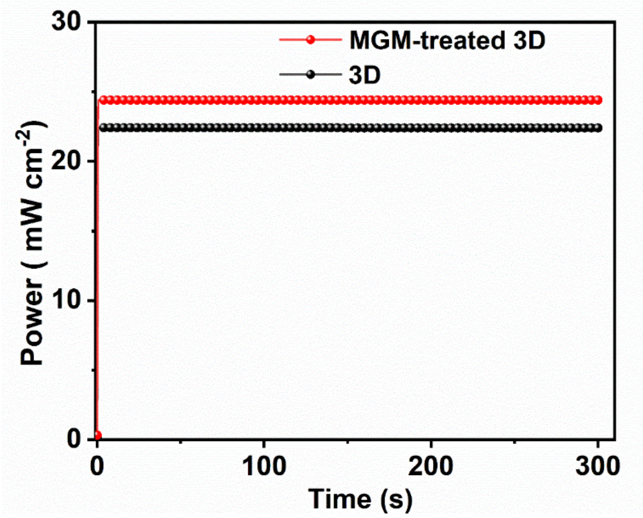

Fig. S11 SPO of 3D film and MGM-treated 3D film-based devices

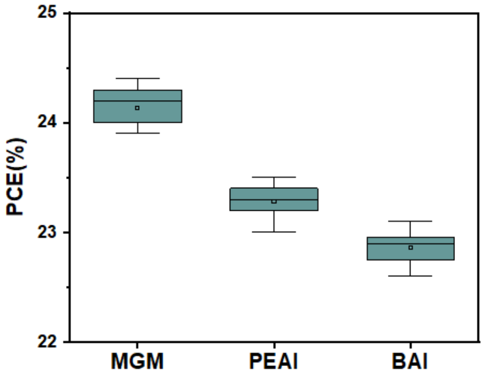

Fig. S12 The statistical PCE distribution of the devices with PEAI, BAI, and MGM treatment (15 devices in each condition)

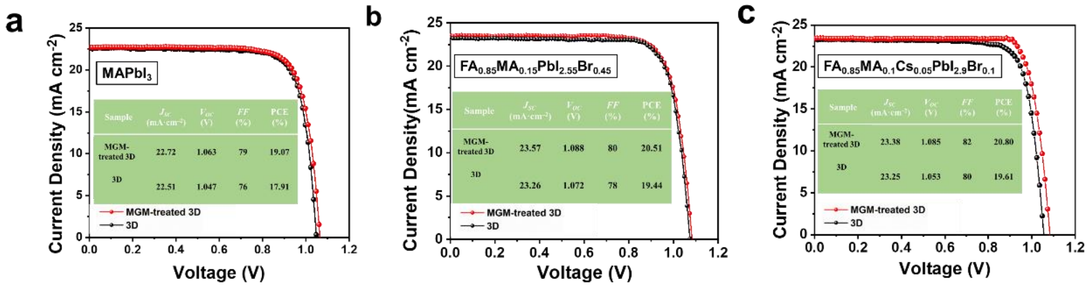

Fig. S13 a *J–V* curves of the n-i-p PSCs with $MAPbI_3$. **b** *J–V* curves of the n-i-p PSCs with $FA_{0.85}MA_{0.15}PbI_{2.55}Br_{0.45}$. **c** *J–V* curves of the n-i-p PSCs with $FA_{0.85}MA_{0.1}Cs_{0.05}PbI_{2.9}Br_{0.1}$

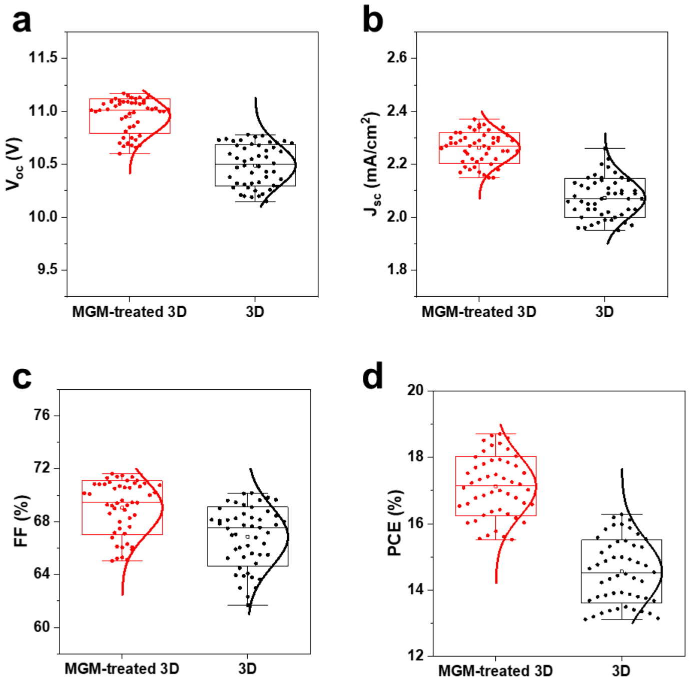

Fig. S14 The statistical box plots on (**a**) V_{OC}, (**b**) J_{SC}, (**c**) FF and (**d**) PCE of the MGM and control modules (50 devices in each condition)

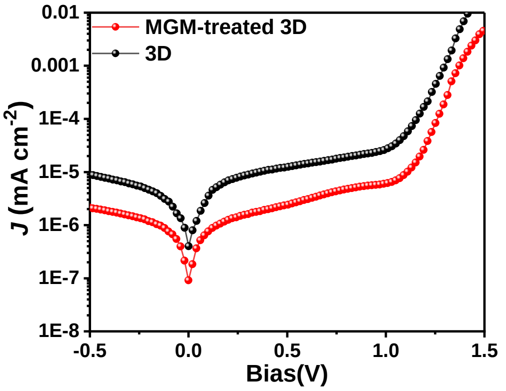

Fig. S15 The dark *J*–*V* of the MGM-treated 3D and 3D film-based devices

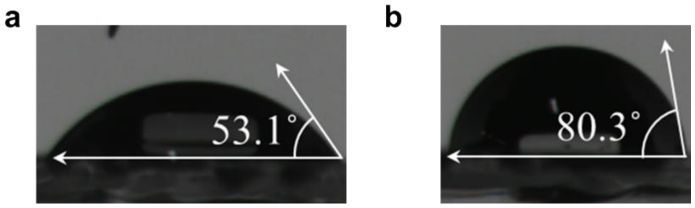

Fig. S16 The water contact angle of **a** the 3D film and **b** MGM-treated 3D film

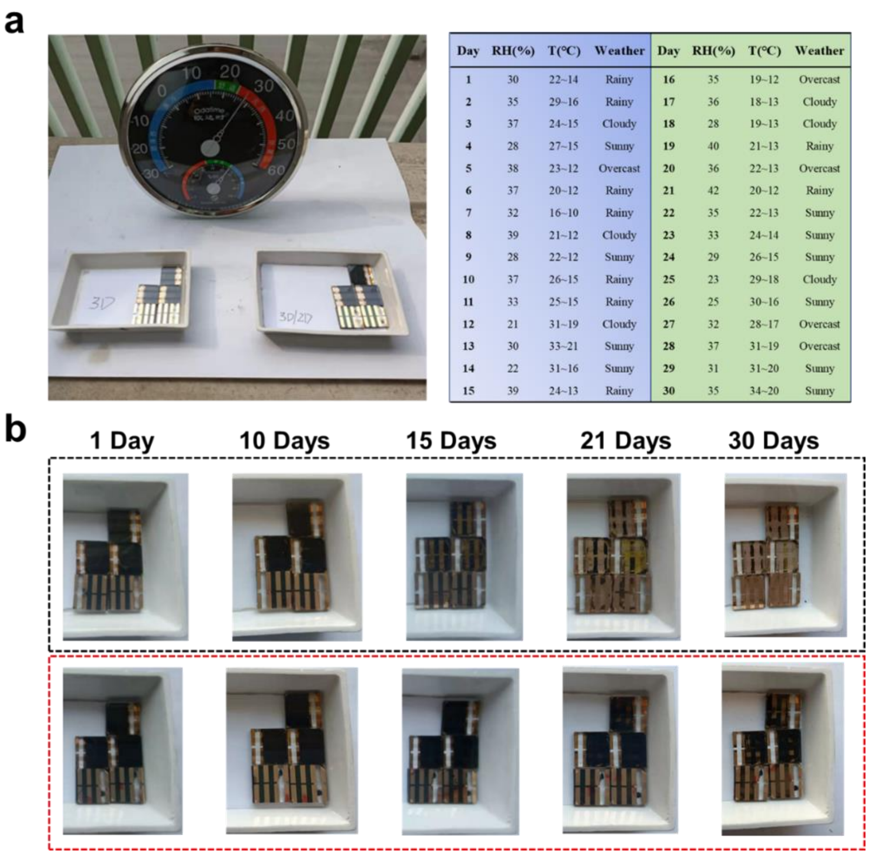

Day	RH(%)	T(°C)	Weather	Day	RH(%)	T(°C)	Weather
1	30	22~14	Rainy	16	35	19~12	Overcast
2	35	29~16	Rainy	17	36	18~13	Cloudy
3	37	24~15	Cloudy	18	28	19~13	Cloudy
4	28	27~15	Sunny	19	40	21~13	Rainy
5	38	23~12	Overcast	20	36	22~13	Overcast
6	37	20~12	Rainy	21	42	20~12	Rainy
7	32	16~10	Rainy	22	35	22~13	Sunny
8	39	21~12	Cloudy	23	33	24~14	Sunny
9	28	22~12	Sunny	24	29	26~15	Sunny
10	37	26~15	Rainy	25	23	29~18	Cloudy
11	33	25~15	Rainy	26	25	30~16	Sunny
12	21	31~19	Cloudy	27	32	28~17	Overcast
13	30	33~21	Sunny	28	37	31~19	Overcast
14	22	31~16	Sunny	29	31	31~20	Sunny
15	39	24~13	Rainy	30	35	34~20	Sunny

Fig. S17 (**a**)The photograph of devices in the natural environment(left) and the real condition of the stability test in the past 30 days(right); (**b**)The photographs of the 3D film (up) and b MGM-treated (down) 3D film-based devices at different stages in the natural environment

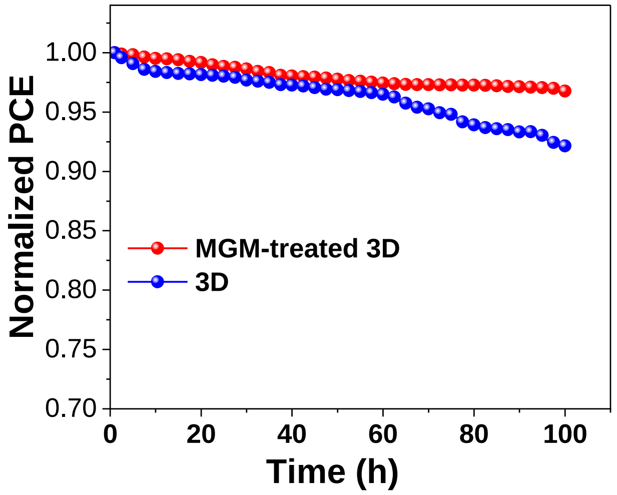

Fig. S18 Operation ISOS-L-1 stability (maximum power point tracking, in Ar, continuous one-sun illumination at ~30 °C) of unencapsulated PSCs based on 3D and MGM-treated 3D film

Table S1 Summarry of the ionic radii of some ammonium cations

**Cations**	MA^+	FA^+	Cs^+	GA^+	BA^+	PEA^+	BDA^{2+}
**Radii(Å)**	2.17	2.53	1.67	2.78	3.93	4.31	4.57

Note: MA^+: methanaminium; FA^+: formamidinium; Cs^+: cesium ;GA^+: guanidinium; BA^+: butylammonium; PEA^+: phenethylammonium; BDA^+: butane-1,4-diammonium;

Table S2 TRPL parameters of amplitudes (A1 and A2) and time constant (τ1 and τ2) for the MCP-treated 3D film and 3D film

**Sample**	τ_1 (μs)	A1 (%)	τ_2 (μs)	A2 (%)	τ_{av} (μs)
**3D**	0.88	42.8	1.8	57.2	1.55
**MGM treatment**	0.35	15.2	5.72	74.8	5.67
**3D/Sprio**	0.98	48.2	0.07	51.8	0.48
**MGM treatment /Sprio**	0.71	5.3	0.04	94.7	0.08

Supplementary Note S1

The curves are fitted using a bi-exponential decay model as Eq. S1:

$$I(\mathrm{t}) = I_0 + A_1 e^{-(t/\tau_1)} + A_2 e^{-(t/\tau_2)} \qquad \text{(S1)}$$

Average lifetime (τ_{av}) is calculated from Eq. S2:

$$\tau_{av} = \frac{A_1 \times \tau_1{}^2 + A_2 \times \tau_2{}^2}{A_1 \times \tau_1 + A_2 \times \tau_2} \qquad \text{(S2)}$$

Table S3 The champion photovoltaic parameters of the 3D film and MGM-treated 3D film-based small area devices (0.12cm^2) under different scan directions

**Sample**	**Scan Direction**	V_{oc} **(V)**	J_{sc} **(mA cm^{-2})**	***FF***	***PCE (%)***
**3D**	Forward	1.13	25.00	0.79	22.3
	Backward	1.13	25.02	0.80	22.6
**MGM treatment**	Forward	1.16	25.21	0.83	24.3
	Backward	1.16	25.23	0.84	24.5

Table S4 The champion photovoltaic parameters of the 3D film and MGM-treated 3D film-based mini-module devices(Aperture area 64 cm^2)

**Sample**	V_{oc}**(V)**	I_{sc}**(mA)**	J_{sc}**(mA/cm^2)**	**FF (%)**	**PCE (%)**
**3D**	10.76	140.03	2.19	69.09	16.3
**MGM treatment**	11.13	150.21	2.35	71.51	18.7
